# Exosome-based topical formulations as adjuncts to fractional Nd: YAG laser for postoperative facial scars

**DOI:** 10.1007/s10103-026-05098-7

**Published:** 2026-11-14

**Authors:** Jun Ho Park, Jei Youn Park, Ji-Ung Park

**Affiliations:** Department of Plastic and Reconstructive Surgery, Seoul National University College of Medicine, SMG-SNU Boramae Medical Center, Seoul, Korea, Republic of

**Keywords:** Nd:YAG, Scar, Exosome, Laser therapy

## Abstract

Commercial topical formulations marketed as exosome skin boosters are increasingly used as adjuncts to laser treatment, but their additional benefit for postoperative facial scars remains uncertain. This randomized controlled trial allocated 150 patients to fractional 1064-nm Nd laser treatment followed by a control hydrogel, a human-derived formulation, or a plant-derived formulation (50 per group). Five treatment sessions were performed at 2-week intervals. Outcomes were assessed at baseline and 2 weeks after the final session using the modified Vancouver Scar Scale (mVSS), patient and observer components of the Patient and Observer Scar Assessment Scale (POSAS), and grayscale intensity (GSI) quantified from Mark-Vu images using ImageJ. Eleven participants were lost to follow-up, leaving 139 participants in the complete-case analysis: 46, 47, and 46 in the respective groups. Both adjunctive formulation groups showed significantly greater reductions in mVSS, observer POSAS, and GSI than the control group. Patient POSAS improved over time but did not demonstrate a significant time × group interaction. No statistically significant difference was detected between the human-derived and plant-derived formulations; equivalence was not established. No serious adverse events were reported during the observed period. Adjunctive formulations were associated with additional short-term improvements in clinician-rated scar outcomes and an exploratory optical measure, without a demonstrated additional patient-reported benefit. These findings apply to the complete formulations and do not establish an exosome-specific effect, sustained scar remodeling, or long-term safety.

**Supplementary Information:**

The online version contains supplementary material available at https://doi.org/10.1007/s10103-026-05098-7.

## Introduction

Postoperative facial scars remain a persistent concern in aesthetic and reconstructive surgery, often leading to psychological distress and dissatisfaction despite technically successful procedures. While current modalities such as fractional laser, pulsed light, and combination toning therapies provide modest improvements in scar appearance, their efficacy is frequently limited by incomplete dermal remodeling, persistent inflammation, and irregular collagen deposition [1–5]. As such, there is increasing interest in biologically active adjuncts that can enhance regenerative outcomes and modulate the wound healing microenvironment [6].

Extracellular vesicles (EVs) are membrane-delimited particles released by cells and have attracted interest as potential adjuncts in tissue repair [4, 5]. Experimental studies suggest that EV-associated proteins, lipids, and nucleic acids may influence inflammatory responses, fibroblast behavior, angiogenesis, and extracellular matrix turnover [5, 6]. However, biological activity varies with the source material, preparation method, and formulation. The term “exosome” denotes a specific biogenesis pathway and cannot be established solely from particle size [7].

Exosomes, nanosized extracellular vesicles (30–150 nm) secreted by various cell types, have emerged as promising candidates in regenerative medicine. Extracellular vesicles can transport proteins, lipids, regulatory RNAs, and signaling molecules involved in inflammation, fibroblast activity, angiogenesis, and extracellular matrix remodeling [4, 5]. Exosomes derived from mesenchymal stem cells have demonstrated efficacy in improving skin elasticity, reducing post-inflammatory hyperpigmentation [6]. However, their widespread clinical use has been limited by high production costs, donor variability, and ethical and regulatory challenges associated with human tissue sourcing [7].

Plant-derived extracellular particle preparations have attracted interest as potential non-animal sources of regenerative adjuncts. Preclinical studies have reported antioxidant, anti-inflammatory, and collagen-related effects, although these findings vary with the plant source and preparation method [8–10]. Their clinical safety and cutaneous penetration require product-specific evaluation and should not be assumed from their plant origin or nanoscale dimensions [11].

Experimental studies have provided additional evidence supporting the investigation of plant-derived preparations for cutaneous repair. Adel et al. reported enhanced epithelial repair, reduced inflammatory cell infiltration, and greater collagen deposition after local injection of a plant-derived preparation in a rat skin wound model [12]. In a rabbit model of laser-induced skin injury, different commercial plant-derived preparations produced formulation-dependent changes in collagen deposition and angiogenesis [13]. These preclinical findings provide a rationale for clinical investigation, although their applicability to topical administration after fractional Nd: YAG treatment remains uncertain.

The plant-derived product evaluated in this study (Exome^®^; Cosmedician Co., Ltd., Busan, Republic of Korea) is a commercial topical formulation described by the manufacturer as containing extracellular vesicle-associated material derived from Centella asiatica and Lavandula angustifolia callus cultures. Although experimental studies of plant-derived preparations provide a rationale for investigating cutaneous applications [14–16], they do not establish the efficacy of this complete commercial product. Because the formulation contains additional ingredients, any observed clinical effect cannot be attributed exclusively to exosomes.

Fractional laser treatment may further facilitate topical delivery by generating spatially confined microthermal treatment zones and transient alterations in cutaneous permeability. Immediate application of biologically active formulations after fractional laser treatment therefore provides a potential strategy for combining laser-induced dermal remodeling with enhanced topical delivery. The 1064-nm Nd: YAG wavelength may be particularly advantageous in Asian patients because of its relatively deep penetration and lower melanin absorption compared with shorter wavelengths, while microlens array technology enables fractional energy delivery with limited disruption of the surrounding epidermis [17].

Accordingly, this randomized controlled study evaluated whether adjunctive application of human-derived or plant-derived commercial topical formulations improved postoperative facial scar outcomes following fractional non-ablative 1064-nm Nd: YAG laser treatment. Laser treatment followed by a control hydrogel was compared with the same laser treatment followed by either formulation. The study evaluated the complete products rather than purified exosomes or an isolated extracellular vesicle-specific effect.

## Materials and methods

### Study design and participants

This prospective, randomized, controlled clinical study enrolled 150 adults aged 20–75 years with clinically visible postoperative facial scars. Participants were randomly allocated to three treatment groups (*n* = 50 per group). Group I received fractional non-ablative 1064-nm Nd: YAG laser treatment followed by a control hydrogel. Groups II and III received the same laser treatment followed by the human-derived and plant-derived topical formulations, respectively.

Participants were randomized using a computer-generated allocation sequence. Allocation concealment was maintained using sequentially numbered, sealed, opaque envelopes prepared by an independent coordinator who was not involved in treatment administration or outcome assessment.

### Eligibility criteria

Inclusion criteria were: (1) a single facial postoperative scar resulting from excision, blepharoplasty, trauma, or flap surgery; (2) scar age between 3 and 8 weeks; (3) Fitzpatrick skin types III–IV; and (4) no prior scar-modifying treatment within 3 months. Exclusion criteria included active dermatologic infection, hypertrophic or keloid scarring tendency, autoimmune disease, diabetes mellitus, isotretinoin use within the preceding 6 months, pregnancy, breastfeeding, or systemic immunosuppressive therapy. Only one representative scar per participant was included in the analysis to avoid within-participant clustering.

### Sample size calculation

An a priori sample size calculation was performed based on preliminary estimates of a mean between-group difference of 1.5 points in mVSS scores with a standard deviation of 1.8, α = 0.05, and 80% statistical power. The calculation indicated that at least 18 participants per group were required. To account for potential attrition and provide sufficient statistical power for secondary analyses, 50 participants were enrolled in each treatment group.

### Plant-derived product formulation

The plant-derived product formulation (Exome^®^; Cosmedician Co., Ltd., Busan, Republic of Korea) was described by the manufacturer as containing extracellular vesicle-associated material derived from aseptically cultivated Centella asiatica and Lavandula angustifolia callus cultures. According to the manufacturer, culture supernatants were processed using centrifugation, filtration, ultracentrifugation, and tangential flow filtration. These manufacturing procedures were not independently verified in the present clinical study.

Particle size distribution and concentration of the PTC raw material were assessed using nanoparticle tracking analysis (NTA; NanoSight, Malvern Panalytical Ltd., Malvern, Worcestershire, UK). Five measurements were analyzed using NanoSight NTA software version 3.4 (build 3.4.003), with a camera level of 16 and a detection threshold of 5 at 24.1–24.3 °C. The merged particle-size distribution showed a mean diameter of 91.1 nm, a modal diameter of 62.9 nm, and a standard deviation of 37.0 nm. The D10, D50, and D90 values were 58.6, 81.2, and 138.9 nm, respectively. The measured particle concentration was (3.18 ± 0.0773) × 10⁸ particles/mL (mean ± standard error across five measurements). After correction for the reported 100-fold sample dilution, the estimated raw-material concentration was 3.18 × 10¹⁰ particles/mL.

These findings support the presence of a measurable nanoscale particle population in the raw material but do not independently establish vesicular identity, purity, or exosomal biogenesis. The raw-material concentration was not interpreted as the particle concentration of the finished topical formulation or the administered particle dose. The product was administered as a complete commercial formulation containing additional ingredients rather than as purified exosomes. Accordingly, the term “plant-derived formulation” is used throughout this manuscript.

### Human-derived product formulation

The human-derived commercial formulation (Exomide^®^; Cosmedician Co., Ltd., Busan, Republic of Korea) was described by the manufacturer as containing material derived from conditioned medium of human adipose-derived mesenchymal stromal cells. According to the manufacturer, the product was prepared using a proprietary processing method and supplied as a topical formulation containing vesicular and non-vesicular components. These manufacturing procedures were not independently verified in the present clinical study.

Particle size distribution and concentration of the human-derived raw material were assessed using nanoparticle tracking analysis (NTA; NanoSight, Malvern Panalytical Ltd., Malvern, Worcestershire, UK). Five measurements were analyzed using NanoSight NTA software version 3.4 (build 3.4.003), with a camera level of 16 and a detection threshold of 5 at 24.1–24.3 °C. The merged particle-size distribution showed a mean diameter of 89.3 nm, a modal diameter of 58.9 nm, and a standard deviation of 34.0 nm. The D10, D50, and D90 values were 56.3, 78.8, and 129.1 nm, respectively. The measured particle concentration was (3.32 ± 0.0486) × 10⁸ particles/mL (mean ± standard error across five measurements). After correction for the reported 100-fold sample dilution, the estimated raw-material concentration was 3.32 × 10¹⁰ particles/mL.

These findings support the presence of a measurable nanoscale particle population in the raw material but do not independently establish vesicular identity, purity, or exosomal biogenesis. The raw-material concentration was not interpreted as the particle concentration of the finished topical formulation or the administered particle dose. The product was administered as a complete commercial formulation containing additional ingredients rather than as purified exosomes. Accordingly, the term “human-derived formulation” is used throughout this manuscript.

### Treatment protocol

Fractional laser treatment was performed using a non-ablative 1064-nm Q-switched Nd: YAG laser system equipped with a microlens array (MLA) fractional handpiece (Pastelle Pro, WONTECH Co., Ltd., Daejeon, Republic of Korea). This device generates uniform non-ablative fractional microthermal zones while preserving the epidermis, enabling controlled dermal targeting for Fitzpatrick skin types III–IV. Each subject underwent five treatment sessions spaced two weeks apart. Each session began with fractional laser treatment using the MLA mode (six passes at a fluence of 11 J/cm2, spot size 3 mm, pulse duration of 10ns and frequency of 3 Hz). One pass was defined as a single complete coverage of the target scar area. Six passes were performed over the scar and its immediate margins, with minimal overlap between adjacent spots. The intended clinical endpoint was mild, uniform erythema without visible epidermal injury.

All three physician evaluators were fully blinded to group allocation. Group I (control) received a sterile, colorless carrageenan-based hydrogel with comparable viscosity to standard post-laser soothing gels (Carragen20 mL; INOT, Republic of Korea), applied immediately after laser treatment. This hydrogel consists primarily of purified water and carrageenan polymer and does not exert any pharmacological or biologically active effects. Groups II and III received the human-derived and plant-derived formulations, respectively, following laser treatment.

Immediately after each session, 1 mL of the assigned topical preparation—control hydrogel in Group I, the human-derived formulation in Group II, or the plant-derived formulation in Group III—was applied evenly over the treated area and gently spread using an aseptic technique. Products were prepared according to the manufacturers’ instructions. All topical preparations (control hydrogel, human-derived formulation, and plant-derived formulation) were identical in appearance, texture, and packaging. Participants were blinded to group allocation. Outcome assessors (physician evaluators) were blinded to treatment assignment throughout the study period. Treating physicians were not involved in outcome assessment. Participants were instructed to use a gentle cleanser, moisturizer, and broad-spectrum sunscreen and to avoid irritating topical products after treatment. No additional laser modes or concomitant scar-directed treatments were used during the study.

### Outcome measures

Clinical outcomes were evaluated at baseline and two weeks after the final treatment session. Subjective scar assessment was performed using the modified Vancouver Scar Scale (mVSS) and the Patient and Observer Scar Assessment Scale (POSAS). All scars were independently evaluated by three blinded third-party plastic surgeons using both tools, and patients also completed the patient portion of the POSAS themselves.

The mVSS assessed six parameters—vascularity, pigmentation, pliability, height, pain, and pruritus—with each item scored on a scale contributing to a total score ranging from 0 (best possible scar) to 16 (worst imaginable scar). The observer component of the POSAS included six criteria: vascularity, pigmentation, thickness, relief, pliability, and surface area. Each parameter was rated from 1 (normal skin) to 10 (worst scar imaginable), yielding a total score range of 6 to 60. The patient component of the POSAS was also composed of six domains: pain, itching, color, stiffness, thickness, and irregularity, each scored from 1 to 10, for a total range of 6 to 60. The mean values from each of the three physician raters and each patient were calculated and used for statistical comparison between groups.

Objective scar evaluation was conducted using the Mark-Vu skin analysis system (Mark-Vu; PSI PLUS, Suwon, Republic of Korea), a non-contact 3D optical imaging device that captures high-resolution topographic and chromatic maps of the facial surface. This system provided quantitative measurements of scar-associated parameters including grayscale intensity (GSI). Grayscale intensity (GSI) obtained from the Mark-Vu skin analysis system was used as an objective optical parameter reflecting surface brightness and textural uniformity of the scar area. Lower GSI values indicate reduced surface reflectance and more homogeneous optical density, which clinically correspond to smoother, less conspicuous scars. The Mark-Vu and similar facial skin analysis systems have been used in previous clinical studies as reproducible tools for quantifying changes in facial skin features before and after treatment. The Mark-Vu skin analysis system enables non-contact and reproducible optical quantification of chromatic and textural skin features, consistent with validated principles used in similar dermatologic imaging platforms [17].

Grayscale intensity (GSI) was quantified from the acquired images using ImageJ software version 1.54 (National Institutes of Health, Bethesda, MD, USA) [18–20]. For each participant, the scar region of interest (ROI) was manually delineated in the baseline and follow-up images using anatomical landmarks to identify corresponding areas. Surrounding unaffected skin was excluded, and the same anatomical scar region was selected at both time points. Images were converted to 8-bit grayscale, and the mean pixel intensity within each ROI was recorded on a scale from 0 to 255. No background normalization, contrast adjustment, or additional image enhancement was performed. ROI selection and analysis were conducted by one investigator blinded to treatment allocation. Changes in mean GSI were compared between assessment points. Lower values indicated lower mean grayscale brightness within the selected scar region. GSI was considered an exploratory optical measure rather than a direct measurement of scar height, surface topography, or collagen remodeling.

### Safety assessment

Adverse events were assessed using participant questioning, and clinical examination at each treatment session and two weeks after the final session. Recorded events were summarized by type, number of affected participants, severity, duration, management, and outcome, where available. Serious adverse events and discontinuations due to adverse events were recorded separately.

### Statistical analysis

Statistical analyses were performed using SPSS version 26.0 (IBM Corp., Armonk, NY, USA). Continuous variables are presented as mean ± standard deviation, and categorical variables as frequency and percentage. Categorical variables were compared using the chi-square test or Fisher’s exact test, as appropriate. When expected cell counts were less than 5, the Fisher–Freeman–Halton exact test was applied for multi-category comparisons. Given the parallel three-group design with two repeated measurements (baseline and post-treatment), continuous outcome variables were analyzed using a two-way repeated-measures ANOVA with time (within-subject factor), group (between-subject factor), and the time × group interaction. Post-hoc pairwise comparisons were conducted using Tukey’s adjustment. Assumptions of normality and homogeneity of variances were examined using the Shapiro–Wilk test and Levene’s test, respectively. When assumptions were violated, results were confirmed using a linear mixed-effects model with random intercepts. Inter-observer reliability for the three physician raters was assessed using the intraclass correlation coefficient (ICC). A p-value < 0.05 was considered statistically significant. Treatment effect sizes were expressed as between-group differences in change from baseline, with corresponding 95% confidence intervals. Change was defined as the post-treatment value minus the baseline value. Negative contrasts indicated a greater reduction in the first-listed group. No equivalence or noninferiority analysis was performed.

## Results

**Fig. 1:**
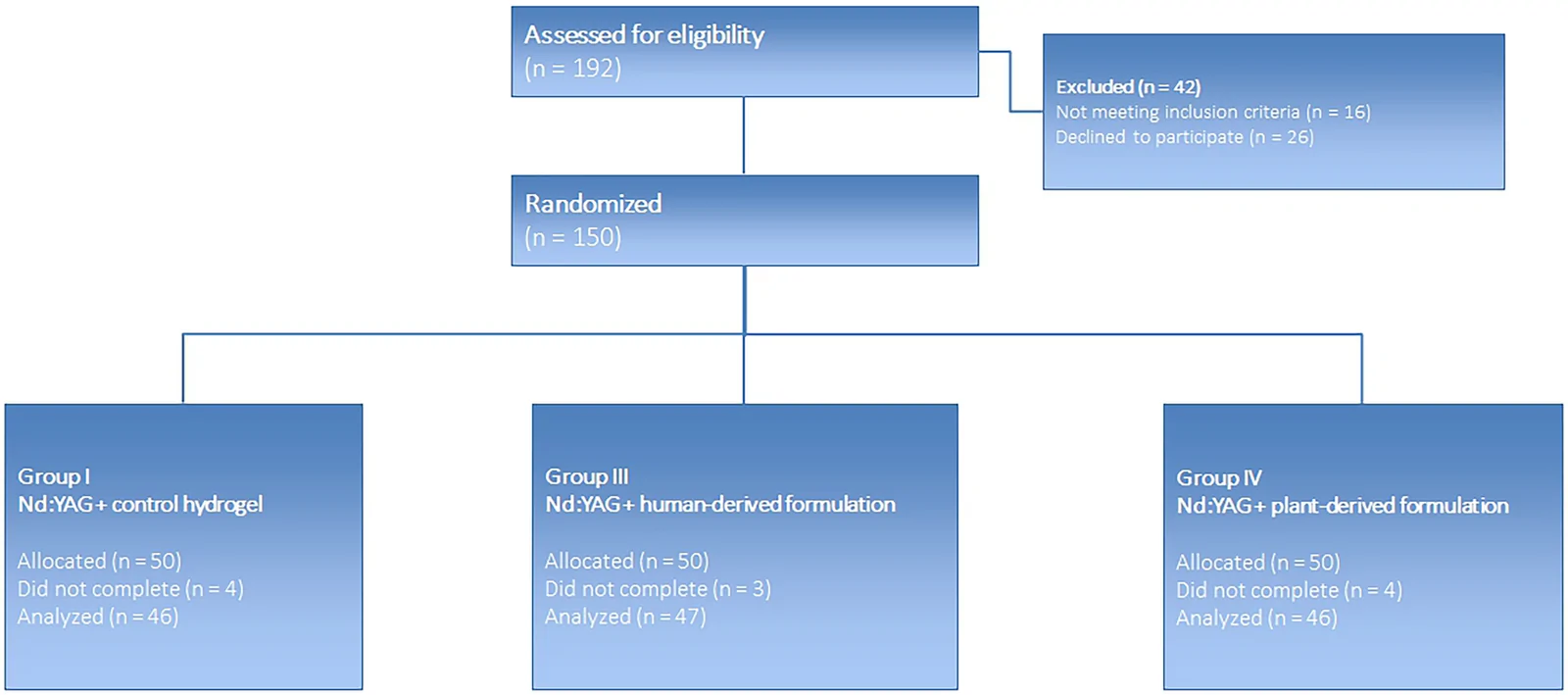
CONSORT flow diagram of participant allocation, follow-up, and analysis. A total of 150 participants were randomized to three groups (50 per group). Eleven participants were lost to follow-up: four in Group I, three in Group II, and four in Group III. The complete-case analysis included 139 participants: 46 in Group I, 47 in Group II, and 46 in Group III. Group I received fractional Nd: YAG laser treatment plus control hydrogel; Group II received laser treatment plus the human-derived formulation; and Group III received laser treatment plus the plant-derived formulation

**Fig. 2:**
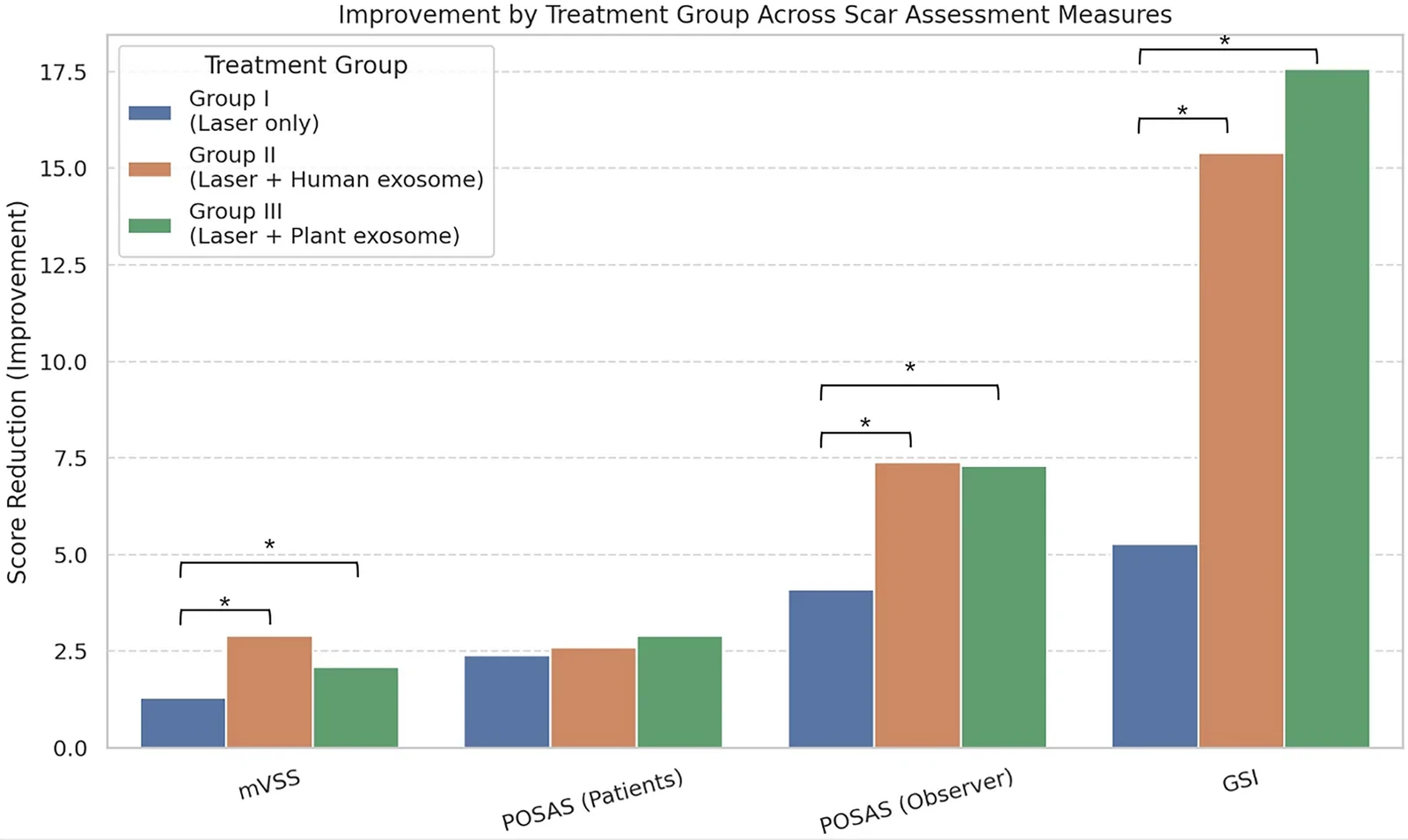
Improvement in scar assessment outcomes across treatment groups. Mean score reductions (post- minus pre-treatment) are shown for modified Vancouver Scar Scale (mVSS), Patient and Observer Scar Assessment Scale (POSAS), and grayscale intensity (GSI). Both formulation-treated groups demonstrated significantly greater improvements compared with laser monotherapy, most notably in observer-rated POSAS and GSI. Asterisks (*) denote statistically significant improvement compared with the laser-only control group (*p* < 0.05)

A total of 150 participants were randomized, with 50 allocated to each of the three treatment groups. Eleven participants were lost to follow-up: four in Group I, three in Group II, and four in Group III. Consequently, 139 participants (92.7%) had complete baseline and final outcome data and were included in the complete-case analysis: 46 in Group I, 47 in Group II, and 46 in Group III. Participant flow is shown in Fig. 1. Baseline characteristics of all randomized participants are presented in Table 1. Outcomes were assessed at baseline and 2 weeks after the fifth treatment session. Repeated-measures ANOVA results for the complete-case population are summarized in Table 2; Fig. 2.

**Table 1:** Baseline characteristics of all randomized participants

Characteristic	Group I	Group II	Group III	*p*-value
Age, years (mean ± SD)	38.6 ± 11.3	45.1 ± 10.8	39.9 ± 11.1	0.21
Sex				0.81
Male	18 (36%)	21 (42%)	18 (36%)	
Female	32 (64%)	29 (58%)	32 (64%)	
Fitzpatrick skin type				0.73
III	36 (72%)	37 (74%)	33 (66%)	
IV	14 (28%)	13 (26%)	17 (34%)	
Scar etiology				0.97
Excision	34 (68%)	34 (68%)	36 (72%)	
Blepharoplasty	5 (10%)	4 (8%)	6 (12%)	
Trauma	6 (12%)	5 (10%)	3 (6%)	
Flap surgery	5 (10%)	7 (14%)	5 (10%)	
Scar age, weeks (mean ± SD)	4.1 ± 2.3	3.8 ± 2.1	4.1 ± 2.4	0.64
Scar location				0.92
Forehead	7 (14%)	5 (10%)	8 (16%)	
Cheek	18 (36%)	21 (42%)	16 (32%)	
Eyelid	10 (20%)	9 (18%)	11 (22%)	
Nose	5 (10%)	7 (14%)	6 (12%)	
Chin	7 (14%)	4 (8%)	8 (16%)	
Perioral	3 (6%)	4 (8%)	1 (2%)	

Values are presented as mean ± SD or n (%)

Baseline characteristics are reported for all randomized participants (50 per group)

The complete-case outcome analysis included 46 participants in Group I, 47 in Group II, and 46 in Group III

P-values were calculated using one-way ANOVA for continuous variables and the chi-square test or Fisher–Freeman–Halton exact test for categorical variables, as appropriate

**Table 2:** Repeated-measures ANOVA results for scar outcomes among the three treatment groups

Outcome	Time effect (*p*)	Group effect (*p*)	Time X Group interaction (*p*)	Tukey-adjusted post-hoc comparisons*
mVSS	< 0.001	0.014	0.009	GII < GI, GIII < GI
POSAS (patient)	< 0.001	0.12	0.18	Not significant†
POSAS (observer)	< 0.001	0.046	0.021	GII < GI, GIII < GI
GSI	< 0.001	0.031	0.007	GII < GI, GIII < GI

* Post-hoc comparisons performed using Tukey’s adjustment (*p* < 0.05)

† Not statistically significant; numerical improvement: GIII > GII> GI

GI: Laser + control hydrogel, GII: Laser + Human derived formulation, GIII: Laser + Plant derived formulation

mVSS: modified Vancouver Scar Scale

POSAS: Patient and Observer Scar Assessment Scale

GSI: Grayscale intensity

For the modified Vancouver Scar Scale (mVSS), a two-way repeated-measures ANOVA revealed significant effects of time (*p* < 0.001) and group (*p* = 0.014), as well as a significant time–group interaction (*p* = 0.009), indicating different patterns of improvement across treatments. Follow-up Tukey testing demonstrated that both adjunctive formulation groups experienced significantly greater reductions in mVSS scores compared with the control group, although the two formulation groups did not significantly differ. Mean scores decreased from 5.4 to 4.1 in the control group, from 5.7 to 2.8 in the human-derived formulation group, and from 5.6 to 3.1 in the plant-derived formulation group.

Patient-reported POSAS scores improved across all groups, with a significant overall time effect (*p* < 0.001), although neither the group effect (*p* = 0.12) nor the interaction effect (*p* = 0.18) reached statistical significance. Mean scores decreased from 13.3 to 10.6 in the plant-derived formulation group, from 14.3 to 12.5 in the human-derived formulation group, and from 13.5 to 11.1 in the control group.

Observer-assessed POSAS scores demonstrated significant time effects (*p* < 0.001) and time–group interaction (*p* = 0.021), indicating that the adjunctive formulation treatments produced greater observer-detectable improvements than laser alone. Scores decreased from 21.8 to 17.7 in the control group, from 22.6 to 15.2 in the human-derived formulation group, and from 23.1 to 15.8 in the plant-derived formulation group. Tukey post-hoc testing confirmed that both formulation groups improved significantly more than the control, with no significant difference between the two formulations. Inter-observer reliability among the three blinded plastic surgeons was excellent (ICC = 0.87–0.91).

**Fig. 3:**
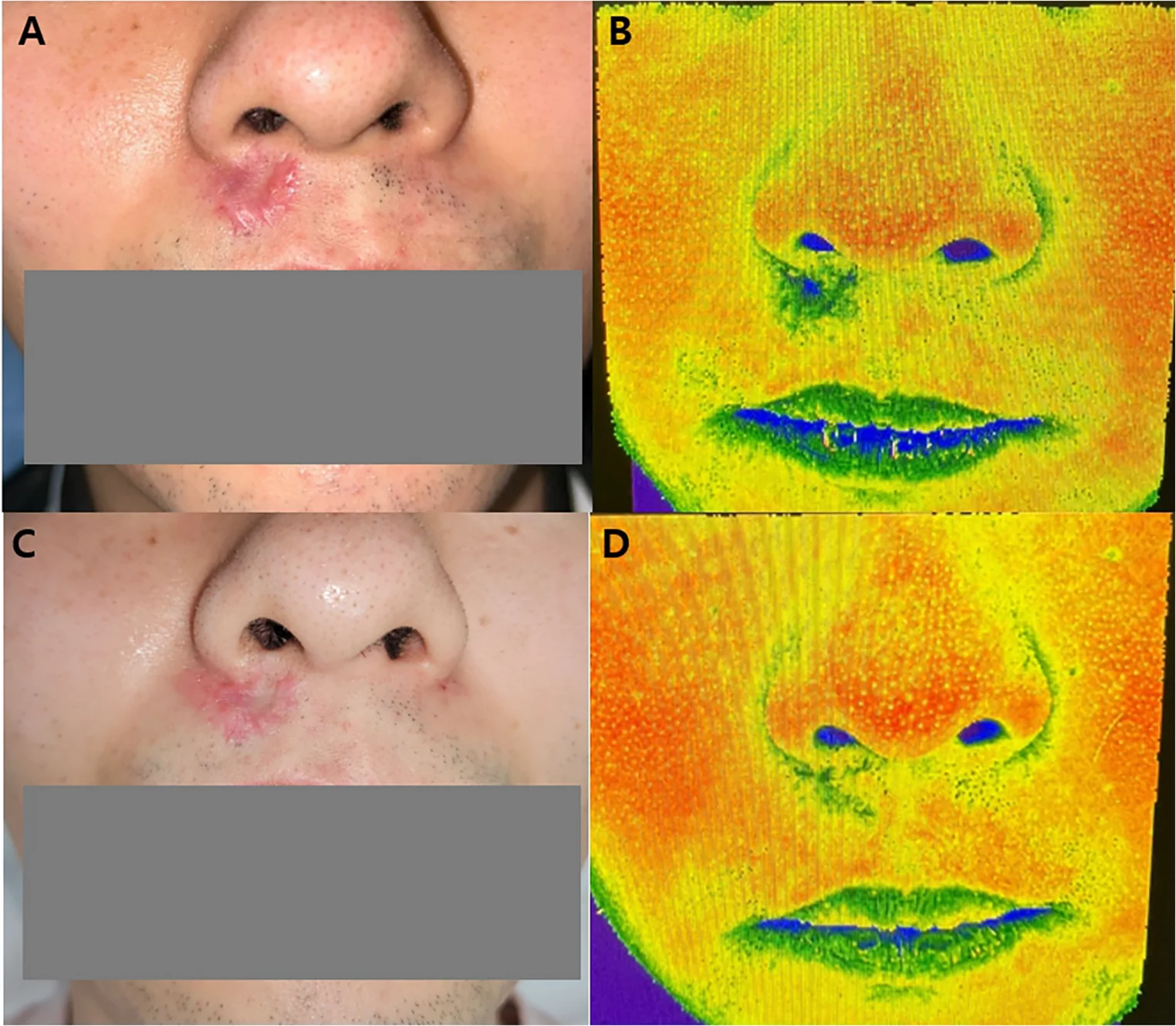
Clinical images of a 29-year-old male patient treated with fractional MLA scar laser and a plant-derived formulation. (**A**) Baseline clinical photograph before treatment. (**B**) Baseline Mark-Vu grayscale intensity analysis (172.64). (**C**) Clinical photograph after five treatment sessions. (**D**) Post-treatment Mark-Vu grayscale intensity analysis (156.39)

**Fig. 4:**
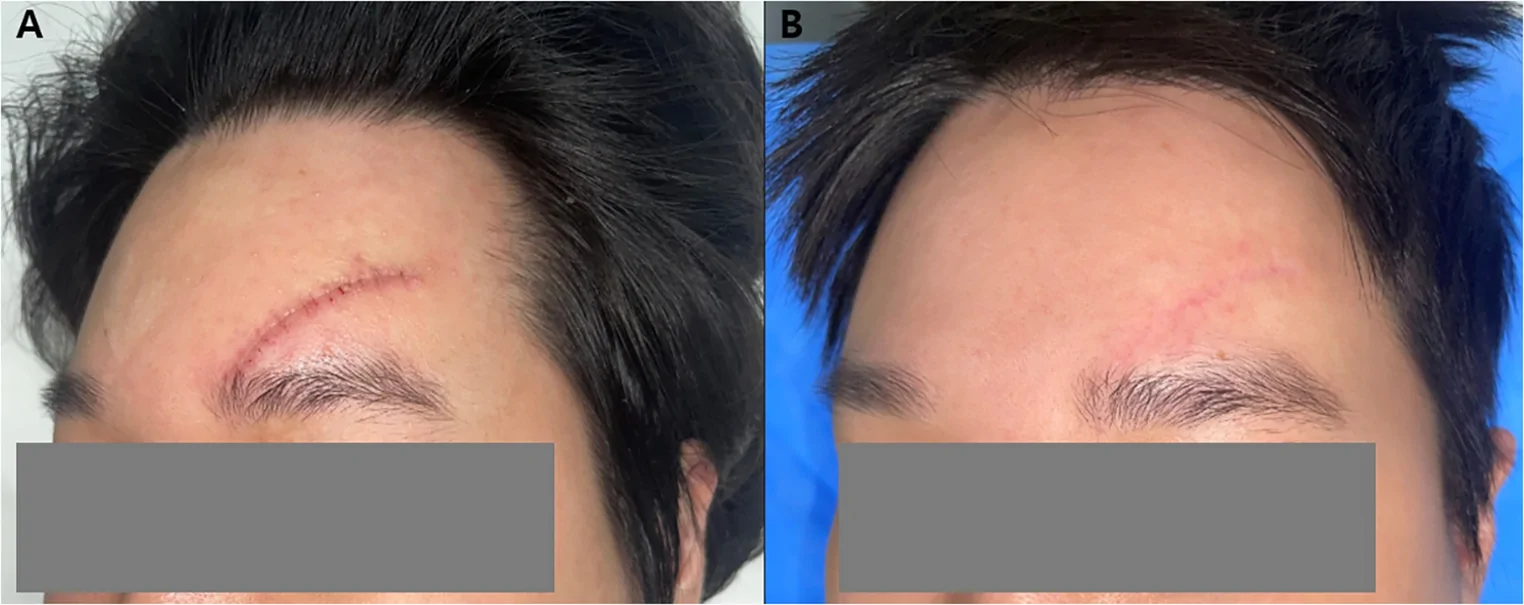
Clinical images of a 34-year-old male patient treated with fractional MLA scar laser and a human-derived formulation. (**A**) Baseline clinical photograph before treatment. (**B**) Clinical photograph after five treatment sessions

**Fig. 5:**
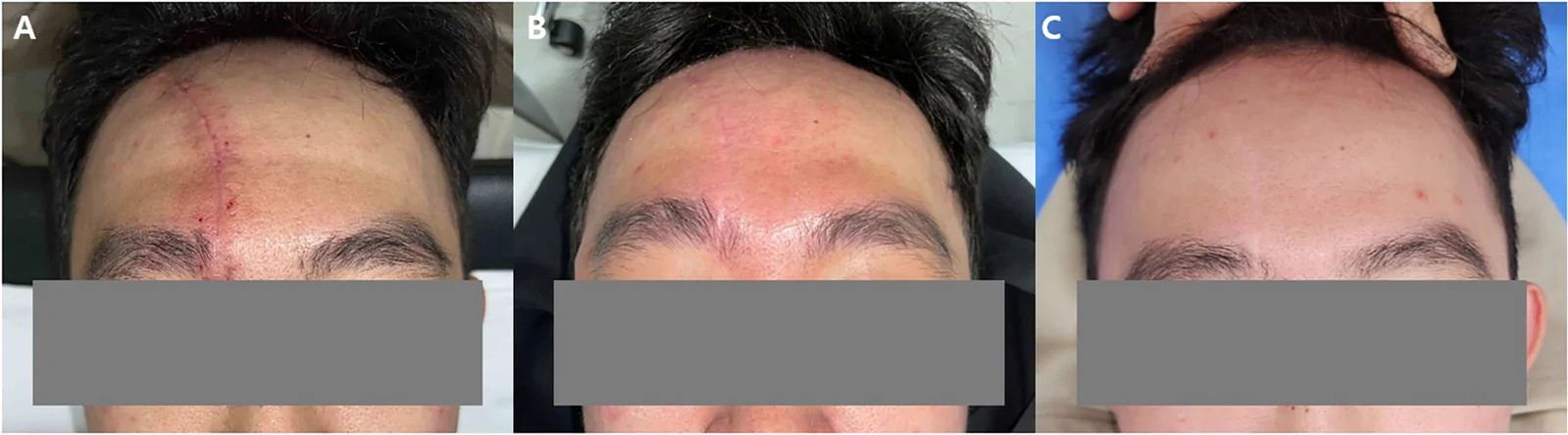
Clinical images of a 24-year-old male patient treated with fractional MLA scar laser and a plant-derived formulation. (**A**) Baseline clinical photograph before treatment. (**B**) Clinical photograph after three treatment sessions. (**C**) Clinical photography after five treatment sessions

Objective optical assessment demonstrated a significant time effect (*p* < 0.001), group effect (*p* = 0.031), and time × group interaction (*p* = 0.007) for GSI. Mean GSI decreased from 166.54 to 161.27 in Group I, from 174.34 to 158.94 in Group II, and from 169.19 to 151.62 in Group III. Both adjunctive formulation groups demonstrated significantly greater reductions than the laser-only group. The difference between the human-derived and plant-derived formulation groups was not statistically significant. Complementary ImageJ-based analysis showed a consistent pattern, including a significant time × group interaction (*p* = 0.016). Between-group differences in change from baseline and their corresponding 95% confidence intervals are presented in Table 3. No statistically significant difference was detected between the human-derived and plant-derived formulations; however, equivalence was not established. Representative clinical and grayscale images from a participant receiving the plant-derived formulation are shown in Fig. 3. Additional clinical photographs from participants receiving the human-derived and plant-derived formulations are presented in Figs. 4 and 5, respectively. These individual examples illustrate clinical appearance and do not replace the group-level outcome analyses.

**Table 3:** Between-group differences in changes in scar outcomes from baseline

Outcome	Comparison	Difference in change	95% CI	Adjusted *p*-value*
mVSS	GII − GI	−1.6	−2.77 to − 0.43	0.004
	GIII − GI	−1.2	−2.37 to − 0.03	0.043
	GIII − GII	0.4	−0.77 to 1.57	0.698
POSAS (patient)	GII − GI	0.6	−0.92 to 2.12	0.620
	GIII − GI	−0.3	−1.82 to 1.22	0.866
	GIII − GII	−0.9	−2.42 to 0.62	0.319
POSAS (observer)	GII − GI	−3.3	−6.34 to − 0.26	0.026
	GIII − GI	−3.2	−6.24 to − 0.16	0.041
	GIII − GII	0.1	−2.94 to 3.14	0.973
GSI	GII − GI	−10.13	−19.48 to − 0.78	0.028
	GIII − GI	−12.30	−21.65 to − 2.95	0.006
	GIII − GII	−2.17	−11.52 to 7.18	0.847

* Pairwise comparisons adjusted using Tukey’s method (*p* < 0.05)

Change: post-treatment − baseline; negative between-group differences indicate a greater reduction in the first-listed group

GI: Laser + control hydrogel; GII: Laser + human-derived formulation; GIII: Laser + plant-derived formulation

CI: confidence interval

mVSS: modified Vancouver Scar Scale

POSAS: Patient and Observer Scar Assessment Scale

GSI: grayscale intensity

No major adverse events occurred during the study. Mild transient burning sensations were reported by two participants in the human-derived formulation group, resolving spontaneously without intervention. All patients completed the full treatment protocol, and adherence to the topical applications was high.

## Discussion

Postoperative scar management remains a significant clinical challenge in aesthetic and reconstructive surgery. Traditional modalities, such as fractional laser resurfacing, radiofrequency micro-needling, and chemical peels, offer limited efficacy due to persistent inflammation, post-inflammatory hyperpigmentation, and inadequate collagen reorganization, particularly in patients with higher Fitzpatrick skin types [21–23]. Although fractional non-ablative lasers can improve dermal texture by stimulating collagen remodeling through controlled thermal injury, their effects are often modest and inconsistent across patient populations [24, 25].

Although fractional CO₂ laser is widely used for laser-assisted delivery by creating ablative microchannels that enhance transdermal penetration of drugs and exosome-based formulations, its use in individuals with Fitzpatrick skin types III–V may be limited by the risk of post-inflammatory hyperpigmentation and prolonged downtime [26]. In contrast, the non-ablative 1064-nm Nd: YAG laser equipped with a microlens array (MLA) generates spatially confined microscopic treatment zones and micro-epidermal disruptions without extensive epidermal ablation. These laser-induced microchannels and dermal photothermal zones can transiently alter the cutaneous barrier and facilitate topical permeation of subsequently applied exosome formulations. Importantly, the relatively low melanin absorption of the 1064-nm wavelength allows deeper energy delivery with a lower risk of pigmentary complications. Non-ablative Nd: YAG MLA has therefore been reported to provide effective scar remodeling with a favorable safety profile in Asian populations, supporting its use as a laser platform for both scar remodeling and laser-assisted topical delivery in postoperative facial scars [27].

Previous experimental studies support the potential of 1064-nm Nd: YAG irradiation to enhance cutaneous delivery under specific treatment conditions. Liu et al. reported that Q-switched 1064-nm Nd irradiation produced micropores in the stratum corneum and enhanced transdermal delivery of glycerol [28]. Lee et al. subsequently demonstrated enhanced cutaneous delivery of cosmeceutical peptides following fractional picosecond- and nanosecond-domain 1064-nm Nd: YAG treatment [29]. These findings provide a mechanistic rationale for investigating topical regenerative adjuncts after fractional Nd: YAG treatment. However, the effects depend on irradiation parameters and the properties of the applied substances; enhanced delivery of small molecules or peptides does not establish equivalent delivery of extracellular vesicles. Because microchannel formation and vesicle penetration were not directly assessed in the present study, enhanced topical delivery remains a proposed mechanism rather than a demonstrated finding.

Among potential regenerative adjuncts, extracellular vesicles have attracted interest because their associated proteins, lipids, and nucleic acids may influence tissue repair [30]. Mesenchymal stem cell (MSC)-derived EVs contain bioactive cargo, including growth factors and regulatory microRNAs, that may modulate fibroblast activity, angiogenesis, and extracellular matrix turnover [31–33]. Preclinical studies and early clinical investigations suggest potential benefits for wound healing and scar improvement [34, 35]. These findings support further investigation of EV-associated formulations as adjuncts to laser treatment, although the contribution of vesicular components cannot be separated from that of other ingredients in the commercial products evaluated here. Clinical translation also remains constrained by manufacturing costs, batch variability, donor-dependent bioactivity, and human tissue-sourcing considerations [36–38].

Formulation-dependent biological effects have also been reported for human-derived preparations. In an experimental study of intact hamster skin, intradermal injection of two human-derived commercial formulations produced different effects on collagen deposition and microvascular density [39]. This finding highlights the importance of product-specific evaluation rather than assuming that formulations marketed as exosome products have interchangeable biological activity. However, the injection route, animal model, and products differed from those used in the present study, so these findings provide indirect biological context rather than evidence of the mechanism underlying our clinical results.

Plant-derived extracellular particle preparations represent another potential source of regenerative adjuncts, with experimental evidence suggesting effects on inflammation, oxidative stress, and tissue repair [8, 11, 14, 40]. Studies of injected plant-derived preparations in skin wound models have reported changes in inflammatory cell infiltration, epithelial repair, collagen deposition, and angiogenesis [12, 13]. A further experimental study found enhanced early regenerative responses when plant-derived preparations were combined with low-level laser therapy in laser-induced skin wounds [41].

These observations support further investigation of combined treatment approaches but should not be directly extrapolated to the present protocol. Injection bypasses the epidermal barrier, and low-level laser therapy differs from fractional Q-switched Nd: YAG irradiation. Furthermore, the tested products and experimental models differed from those used here. Accordingly, these studies do not establish enhanced topical vesicle delivery, product-specific clinical efficacy, or long-term safety for the formulations evaluated in our trial.

In this randomized clinical trial, both adjunctive commercial formulations were associated with greater short-term reductions in mVSS, observer-rated POSAS, and grayscale intensity than laser treatment with a control hydrogel. Patient-reported POSAS did not demonstrate a significant additional treatment benefit. These findings apply to the complete formulations and do not establish that extracellular vesicles were responsible for the observed differences. No statistically significant differences were detected between the human-derived and plant-derived formulations. However, equivalence was not established because no equivalence or noninferiority margin was prespecified.

The discrepancy between observer-rated and patient-reported outcomes warrants careful consideration. Although mVSS and observer POSAS favored the adjunctive formulation groups, patient POSAS showed no significant time × group interaction. Thus, the clinician-rated improvements were not accompanied by a demonstrated additional patient-perceived benefit during the observed period. Observer and patient POSAS assess partly different aspects of scarring, and clinician-detectable changes may not translate into noticeable improvements in symptoms or appearance for patients. The relatively low baseline patient POSAS scores and short follow-up may have limited the detection of additional patient-perceived benefits, although these explanations were not formally tested. Accordingly, the clinician-rated and grayscale findings should be interpreted alongside the absence of a significant patient-reported treatment effect. The patient-perceived value of the adjunctive formulations remains uncertain.

Experimental literature suggests that EV-associated cargo may influence inflammatory signaling, fibroblast responses, and extracellular matrix turnover [9, 15, 42, 43]. However, these mechanisms were not measured in the present study and cannot be assigned to the commercial formulations on the basis of the clinical findings alone. Non-vesicular ingredients and vehicle-related effects may also have contributed to the observed outcomes. Because the study did not include matched vehicles or EV-depleted preparations, the relative contributions of vesicular and non-vesicular components remain uncertain [44].

Our objective assessments using Mark-Vu imaging and ImageJ pixel analysis confirmed optical and structural improvements in scar appearance, correlating with improved dermal homogenization and texture. This approach enabled a robust, quantitative evaluation of surface irregularities and provided a more nuanced interpretation of treatment outcomes beyond subjective scoring scales.

This study has several limitations. First, the final assessment was performed only 2 weeks after the fifth treatment session. The findings therefore reflect short-term changes in scar scores and optical appearance rather than sustained scar remodeling. This follow-up period does not establish whether the observed differences persist after treatment cessation or translate into long-term collagen maturation. Because the scars were relatively recent at enrollment, natural scar maturation may also have contributed to improvement across all groups. Delayed adverse effects could not be adequately assessed. Longer-term follow-up, preferably for at least 6 months after the final treatment, is needed to evaluate the durability of benefit and longer-term safety. Second, grayscale intensity (GSI) should be interpreted as an indirect optical measure rather than a validated histologic surrogate for dermal remodeling. Although GSI reflects optical changes rather than direct histologic alterations, its use as a quantitative imaging parameter in clinical scar assessment has been supported in previous studies [19, 20]. Third, although adherence to post-laser exosome application was monitored, variations in patient compliance may have introduced subtle bias. Fourth, although scar locations were comparable among groups at baseline, randomization was not stratified by anatomical site, which may have influenced treatment response. Fifth, the commercial formulations contained additional ingredients, and characterization of the raw materials did not establish the identity, purity, or particle dose of the finished products. The study therefore cannot distinguish EV-associated effects from those of non-vesicular ingredients or formulation vehicles.

Finally, because the study population consisted exclusively of East Asian participants with Fitzpatrick skin types III–IV, the generalizability of these findings to other ethnic groups and skin phototypes remains uncertain. Future multicenter studies incorporating longer follow-up, broader patient populations, and histologic or molecular endpoints are warranted.

## Conclusions

Adjunctive human-derived and plant-derived commercial topical formulations following fractional non-ablative 1064-nm Nd: YAG laser treatment were associated with greater short-term improvements in clinician-rated scar outcomes and an exploratory optical measure than laser treatment with a control hydrogel. Patient-reported POSAS did not demonstrate an additional benefit. No statistically significant difference was detected between the formulations, but equivalence was not established. These findings apply to the complete products and do not establish an exosome-specific effect, sustained scar remodeling, or long-term safety.

## Supplementary Information

Below is the link to the electronic supplementary material.

Supplementary Material 1

## Data Availability

No datasets were generated or analysed during the current study.
