# Supplementary material for: Exosome-based topical formulations as adjuncts to fractional Nd: YAG laser for postoperative facial scars
Source: Lasers Med Sci. 2026 Nov 14;41(1):296. doi: 10.1007/s10103-026-05098-7 (PMC13769748; doi:10.1007/s10103-026-05098-7)
Supplement: Supplementary file 1 [file 10103_2026_5098_MOESM1_ESM.docx]

Supplementary Table 1. Mean scar outcomes per group (baseline vs. post-treatment)

|  | Group I | | Group II | Group III |
| --- | --- | --- | --- | --- |
| Mean mVSS | |  |  |  |
| Pre | | 5.4 | 5.7 | 5.6 |
| Post | | 4.1 | 2.8 | 3.1 |
| Improvement | | 1.3 | 2.9 | 2.5 |
| Mean POSAS (Patients) | |  |  |  |
| Pre | | 13.5 | 14.3 | 13.3 |
| Post | | 11.1 | 12.5 | 10.6 |
| Improvement | | 2.4 | 1.8 | 2.7 |
| Mean POSAS (Observer) | |  |  |  |
| Pre | | 21.8 | 22.6 | 23.1 |
| Post | | 17.7 | 15.2 | 15.8 |
| Improvement | | 4.1 | 7.4 | 7.3 |
| GSI | |  |  |  |
| Pre | | 166.54 | 174.34 | 169.19 |
| Post | | 161.27 | 158.94 | 151.62 |
| Improvement | | 5.27 | 15.40 | 17.57 |

mVSS: modified Vancouver Scar Scale

POSAS: Patient and Observer Scar Assessment Scale

GSI: Grayscale intensity
